# 

**Published:** 1887-07

**Authors:** 


### BOOKS AND PAMPHLETS RECEIVED.

JAHRESBERICHT DER K. CENTRAL-THIERARZNEI-SCHULE IN MUNCHEN 1885-86. Leipzig, 1887.

CHICAGO VETERINARY COLLEGE. Prospectus for Session 1887-88.

ANNUAL ANNOUNCEMENT OF THE NEW YORK COLLEGE OF VETERINARY SURGEONS AND SCHOOL OF COMPARATIVE MEDICINE, 1887-88.

JOHNS HOPKINS UNIVERSITY CIRCULARS. Baltimore.

THE JOHNS HOPKINS UNIVERSITY REGISTER, 1886-87.

THE CORNELL REGISTER, 1886-87. Ithica, N. Y.

ANNUAL ANNOUNCEMENT OF THE LONG ISLAND COLLEGE HOSPITAL. Brooklyn, N. Y., 1887.

ANNUAL REPORT OF THE BOARD OF MANAGERS OF THE NORTH-EASTERN DISPENSARY IN THE CITY OF NEW YORK. 1887.

PERSISTENT PAIN AFTER ABDOMINAL SECTION. By James B. Hunter, M. D. (Reprint from Gynecological Transactions, 1886.)

THE PAST, PRESENT AND FUTURE TREATMENT OF HOMŒOPATHY ELECTICISM AND KINDRED DELUSIONS. By Henry I. Bowditch, M.D., Boston, 1887.

REPORT OF THE COUNCIL OF THE ZOOLOGICAL SOCIETY OF LONDON FOR THE YEAR 1886.

ANNUAL REPORT OF THE AMERICAN MUSEUM OF NATURAL HISTORY FOR THE YEAR 1886-87.

ANNUAL REPORT OF THE BOARD OF REGENTS OF THE SMITHSONIAN INSTITUTION. PART I. Washington, 1886.

CONTRIBUTIONS TO THE ANATOMY OF GEOCOCCYX CALIFORNIANUS AND ADDITIONAL NOTES UPON THE ANATOMY OF THE TROCHILI, CAPRIMULGI AND CYPSELIDÆ. By R. W. Shufeldt, M. D., C. M. Z. S. (Reprint from the Proceedings of the London Zoological Society, 1887.)

CAPTAIN GLAZIER AND HIS LAKE. An inquiry into the history and progress at the head waters of the Mississippi. Ivison, Blakeman & Co., New York.

The following foreign publications are to hand :

The Veterinarian, June, 1887. The Veterinary Journal, June, 1887. The Quarterly Journal of Veterinary Science in India, April, 1887. Gaceta Medico-Veterinaria, June, 1887. La Cronica Medica, May, 1887. Giornale di Anatomia Fisiologia e Patologia degli Animali, April, 1887. Medico Veterinario, April, 1887. Bollettino Scientifico, March, 1887. La Clinica Veterinaria, June, 1887. Le Progress Medical, June, 1887. Annals et Bulletin Societie de Medicine de Gand, June, 1887. Revue Veterinaire, June, 1887. Der Zologische Garten, March, 1887. Wochenscrift fur Thierheilkunde und Viehzucht, May, 1887. Schweizer-Archiv fur Thierheilkunde, June, 1887. Archiv fur Wissenschaftliche und Praktische Thierheilkunde Band 13, Heft 3. Monatsschrift des Vereines der Theirarzte in Oesterreich, June, 1887. Tidskrift fur Veterinar-Medicin och Husdjurss-Kotsel, April, 1887. Bladen uitgegeven door de Vereeniging tot Bevordering van Veeartsenijkunde in Nederlandsch-Indie Deel 11, Afl 1. The Archives of Veterinary Science, St. Petersburg, June, 1887.

